# Causes of Death among Children Aged 5 to 14 Years Old from 2008 to 2013 in Kersa Health and Demographic Surveillance System (Kersa HDSS), Ethiopia

**DOI:** 10.1371/journal.pone.0151929

**Published:** 2016-06-15

**Authors:** Melkamu Dedefo, Desalew Zelalem, Biniyam Eskinder, Nega Assefa, Wondimye Ashenafi, Negga Baraki, Melake Damena Tesfatsion, Lemessa Oljira, Ashenafi Haile

**Affiliations:** 1 Kersa Health and Demographic Surveillance System (Kersa HDSS), Harar Ethiopia; 2 Haramaya University, College of Computing and Informatics, Department of Statistics, Dire Dawa, Ethiopia; 3 Haramaya University, College of Health and Medical Sciences, Harar, Ethiopia; 4 Centers for Disease Control and Prevention (CDC-Eth), Addis Ababa, Ethiopia; Institut Pasteur, FRANCE

## Abstract

**Background:**

The global burden of mortality among children is still very huge though its trend has started declining following the improvements in the living standard. It presents serious challenges to the well-being of children in many African countries. Today, Sub-Saharan Africa alone accounts for about 50% of global child mortality. The overall objective of this study was to determine the magnitude and distribution of causes of death among children aged 5 to 14 year olds in the population of Kersa HDSS using verbal autopsy method for the period 2008 to 2013.

**Methods:**

Kersa Health and Demographic Surveillance System(Kersa HDSS) was established in September 2007. The center consists of 10 rural and 2 urban kebeles which were selected randomly from 38 kebeles in the district. Thus this study was conducted in Kersa HDSS and data was taken from Kersa HDSS database. The study population included all children aged 5 to 14 years registered during the period of 2008 to 2013 in Kersa HDSS using age specific VA questionnaires. Data were extracted from SPSS database and analyzed using STATA.

**Results:**

A total of 229 deaths were recorded over the period of six years with a crude death rate of 219.6 per 100,000 population of this age group over the study period. This death rate was 217.5 and 221.5 per 100,000 populations for females and males, respectively. 75% of deaths took place at home. The study identified severe malnutrition(33.9%), intestinal infectious diseases(13.8%) and acute lower respiratory infections(9.2%) to be the three most leading causes of death. In broad causes of death classification, injuries have been found to be the second most cause of death next to communicable diseases(56.3%) attributing to 13.1% of the total deaths.

**Conclusion and Recommendation:**

In specific causes of death classification severe malnutrition, intestinal infectious diseases and acute lower respiratory infections were the three leading causes of death where, in broad causes of death communicable diseases and injuries were among the leading causes of death. Hence, concerned bodies should take measures to avert the situation of mortality from these causes of death and further inferential analysis into the prevention and management of infectious diseases should also be taken.

## Introduction

The global burden of mortality among children is very huge though its trend has started declining following the improvements in the living standard which was caused by socio-economic development[1–3]. Its level varies by regions of the world in which the developed countries experiencing lower mortalities compared to the developing world[3].

Burdens of childhood mortality can be clearly understood when causes of death are reliably ascribed, and some causes may be more susceptible to reduction than others where the combination of causes varies noticeably between different settings, as well as between age groups[4, 5]. Information about cause of death is needed to prioritize interventions and plan for their delivery, to ascertain the effectiveness of disease specific interventions, and to assess trends in disease burden in relation to national and international aims[6].

Developing countries experience more than three fourth of deaths in the world, but the causes of these deaths are not well documented, and in many of these countries, vital registration systems are non-existent or at best rudimentary, and even when deaths are registered, data on the cause of death, which is an important step toward improving public health, are lacking[7–12]. In such settings with poor or no routine death certification, a significant number of studies have shown that conducting verbal autopsy (VA) is the best available approach that can be used to generate reasonable population level estimates and in obtaining the necessary empirical information on the cause of deaths[9, 11–19].

A review of literature done in sub Saharan Africa has suggested that at ages 5 to 14 years malaria, diarrheal and malnutrition were the leading causes of death among school age children[20]. Similarly, a study on cause specific childhood mortality in Africa and Asia from International Network for Demographic Evaluation of Populations and Their Health (INDEPTH) surveillance system sites for the age group 5 to 14 years showed that external causes, malaria and non-communicable diseases were among the main cause of death in the region[5]. And another study done in Addis Ababa on patterns of mortality showed that children of age below 15 were 16 times more likely to die from communicable, prenatal and nutritional conditions compared to their elders[21].

In general, studies focusing on child mortality (specifically for children aged 5 to 14), are scanty. It is this fact that motivated the initiation of the current study and the report focuses on the causes of death among children aged 5 to 14 as well as the socio demographic characteristics of them. The objective of this study was to determine the patterns and distribution of causes of death of children among age group of 5 to 14 years in the population of Kersa HDSS, Ethiopia using verbal autopsy data from 2008 to 2013.

## Methods

Kersa HDSS is located in Kersa district between *41*°*40*^*”*^*0*^*’*^ and *41*°*57*^*”*^*30*^*’*^ E and *09*°*15*^*”*^*15*^*’*^ and *09*°*29*^*”*^*15*^*’*^ N and in terms of ecology it covers highland (7%), midland (91%) and lowland (2%) agro-climatic zones[22, 23]. The elevation ranges from 1400 to 3200 meters above sea level, the monthly minimum average temperature is 12.0°C and the maximum average temperature is 24.2°C, and the monthly average rainfall is 65 mm[22, 24]. According to the 2007 national census, the district has a total population of 172,626 of whom 6.9% are urban dwellers, and a population density of 372 people per square kilometer[25]. Agriculture is the main economic activity of the population. Cereal crops such as sorghum, maize, wheat, barley, oats and teff and cash crops such as field peas, lentils, ground nuts, linseeds, chat, coffee, etc are main agricultural products of the district. In 2007 the district had one health center and eight clinic/health posts. The potential health service coverage was 56%. About 7.7% of the total population had access to safe drinking water (22.1% of the urban, 6.9% of the rural)[22, 26].

The Kersa Demographic Surveillance and Health Research System (Kersa HDSS) was established in September 2007 by Haramaya University, during the same time 12 kebeles(smaller administrative region in Ethiopia) were randomly selected out of 38 kebeles in the district (10 rural and 2 urban)[22]. The center was established with objectives of: collecting longitudinal population based data on demographic, health and environmental issues to be used for planning and evaluating heath intervention programs, enhance research culture in the teaching learning process, serve as center of research, disseminate research findings and, advocate utilization of research findings to improve health service delivery[22]. Kersa HDSS is currently an INDEPTH network member.

Ethical clearance was secured from Institutional Health Research Ethics Review committee of Haramaya University and then approved by Ethiopian Public Health Association (EPHA) and CDC Atlanta and permission was obtained from local authorities. Informed verbal consent was obtained from head of the family or an eligible adult in the family. This verbal consent was documented in English and local languages “Afaan Oromo and Amharic”.

After obtaining ethical clearance and conducting initial census in September 2007, vital events (births, deaths, migration, marriages, etc.) and health related data have been monitored on continuous basis. Data on cause of death for the deceased is also collected on continuous basis using death registration forms by interviewing the close relatives or caregivers of the deceased. Field enumerators and or local guides use death registration forms to collect information on the deceased. Then, these completed forms were transferred to VA interviewers. Address, name, sex, and date of death were the minimum set of variables included in the registration forms.

In Kersa HDSS, age specific VA questionnaires (5 to 14 years of age) were modified and adopted from Verbal Autopsy Interviewer’s Manual, Sample Vital Registration with Verbal Autopsy, MEASURE Evaluation Project and US Census Bureau. The questionnaire was translated into local languages, Afaan Oromo and Amharic and back translated to English to check for consistency. The translated VA questionnaires were used to identify information including age, sex, place of death, cause of death based on narrative history of respondents; symptom duration checklist, health services used in the period before death, and history of previously known medical conditions. The questions contained in the symptom duration checklist were arranged systematically around anatomical systems, and were intended to be as informative as possible in leading to a diagnosis of probable cause of death.

In the surveillance system there are permanently hired field enumerators who are responsible for recording and reporting deaths that occur in the Kebeles (the smallest administrative unit) of the surveillance system. Field data collectors and local guides assisted the VA interviewers (VAI) by making appointments to meet with bereaved families. The VA interviewers were 4–6 in number throughout the study period and they, at least completed high school education before recruited to conduct the VA interview. They were trained on how to contact respondents, when to interview and complete the questionnaires. The training curriculum included sessions on discussion of individual symptoms, and their description in local language for easy recognition by the respondents and demonstration of interviewing techniques by surveillance team. The VAIs were informed about new deaths by the field enumerators and local guides and conduct verbal autopsy interviews within one to three months after death considering the local mourning period. VAIs reported to the surveillance team using a prepared activity reporting format weekly.

Two physicians independently reviewed the completed VA questionnaires and assigned diagnosis using ICD-10 code and title and then converted into VA code and title. The agreements between the two physician diagnoses were checked by the members of the surveillance team. When there are disagreements in diagnosis, a third physician was assigned to review the cases and a final diagnosis was assigned based on the agreement between any of the two physicians. If the three physicians assigned different diagnosis, the case was labeled as undetermined or undecided.

Seven surveillance team members from the University coordinated the field activities of the VAIs and were responsible for making sure that the field operations run smoothly and efficiently and also give supervisory support to VA interviewers. The team is responsible for the data management, analysis and report writing. During the course of the fieldwork, supervisors and surveillance team continually visited the site to check on the progress and sort out problems that might have been encountered by enumerators. Regular meeting with enumerators were held in which enumerators and supervisors discussed various issues related to the progress of fieldwork, and completed questionnaires were examined for consistency and plausibility with the surveillance team. Periodically, approximately 5% of households were selected randomly for re-interviews for verification purposes. All surveillance team including data collectors, supervisors, VAI, physician VA reviewers and surveillance team met on a regular basis to discuss of the activities and make corrections on filled questionnaire before the questionnaires are compiled and sent to data entry.

Data coding and cleaning were done and entered into a database by trained data encoders using SPSS and transferred into STATA for analysis. Backup copies of the electronic files of the data are taken regularly. Physician reviews and assigned diagnosis were coded using VA coding system from ICD-10.

## Results

Verbal autopsy interviews were conducted for a total of 229 deaths of children aged 5 to 14 years out of a total 3030 deaths during the year 2008 to 2013. Over the entire period of the study time a crude death rate was 219.6 per 100,000 population of children 5 to 14 years age group and this death rate was 217.5 and 221.5 per 100,000 populations for females and males, respectively. From Table 1 we can see that, of the total diseased, 216(94%) were from rural and 13(6%) were from urban part of the study area. Among the diseased, 119(52%) of them reported to be males and 110(48%) were females. One in two of the diseased had no formal schooling, where six out of thirteen had followed primary education. About 86% of deaths occurred outside health institutions and three fourth of the diseased were reported to be at home.

**Table 1:** Socio-Demographic Characteristics and place of death of the deceased by year among 5 to14 year olds, Kersa HDSS, 2008–2013.

Characteristics	Year													
	2008		2009		2010		2011		2012		2013		Total	
Residence	N	%	N	%	N	%	N	%	N	%	N	%	N	%
**Urban**	1	3.45	2	7.14	2	6.25	2	3.03	5	14.71	1	2.50	13	5.68
**Rural**	28	96.55	26	92.86	30	93.75	64	96.97	29	85.29	39	97.50	216	94.32
**Total**	29	100.00	28	100.00	32	100.00	66	100.00	34	100.00	40	100.00	229	100.00
**Sex**														
**Male**	17	58.62	14	50.00	16	50.00	34	51.52	19	55.88	19	47.50	119	51.97
**Female**	12	41.38	14	50.00	16	50.00	32	48.48	15	44.12	21	52.50	110	48.03
**Total**	29	100.00	28	100.00	32	100.00	66	100.00	34	100.00	40	100.00	229	100.00
**Education**														
**Illiterate**	1	12.50	10	52.63	12	60.00	23	60.53	8	35.00	12	38.71	66	49.62
**1–4**	6	75.00	8	42.11	8	40.00	15	39.47	14	61.00	11	35.48	62	46.62
**5–8**	1	12.50	1	5.26	0	0.00	0	0.00	1	4.00	0	0.00	3	2.26
**9–10**	0	0.00	0	0.00	0	0.00	0	0.00	0	0.00	8	25.81	2	1.50
**Total**	8	100.00	19	100.00	20	100.00	38	100.00	23	100.00	31	100.00	133	100.00
**Place of death**														
**Hospital**	3	10.35	4	14.28	2	6.25	5	7.57	8	23.53	4	10	26	11.35
**Health Center**	2	6.89	0	0	0	0	1	1.51	1	2.94	1	2.5	5	2.18
**Other Health Institutions**	1	3.45	0	0	0	0	0	0	0	0	0	0	1	0.44
**Home**	21	72.41	19	67.86	27	84.38	55	83.33	19	55.88	30	75	171	74.67
**Other place**	2	6.89	5	17.86	3	9.37	5	7.58	6	17.65	5	12.5	26	11.35
**Total**	29	100	28	100	32	100	66	100	34	100	40	100	229	100

The causes of death in the study population (children aged 5–14 years) was specified for 191(83%) cases and 38(17%) of the cases were remained unspecified. In this report we focus only on the specified causes of death. Infectious and parasitic diseases were the leading cause of death accounting for about 31% of deaths, followed by malnutrition and endocrine with about 26%. Throughout the study period infectious and parasitic and nutritional and endocrine disorders were the two leading broad causes of death among this age group. Furthermore Table 2 shows that major causes of death among children aged 5 to 14 is due to communicable diseases(56.3%) followed by unspecified cause of death(16.6%) and injuries(13%). Death from injuries has shown increment with little fluctuation over the study period.

**Table 2:** Broad causes of death by year among 5–14 year old children, Kersa HDSS, 2008–2013.

No	Causes of Death	Year												Total		
		2008		2009		2010		2011		2012		2013				
		N	%	N	%	N	%	N	%	N	%	N	%	N	%	Sum %
**1**	**Communicable diseases**															56.33
	Infectious and parasitic	8	27.59	6	21.43	12	37.50	23	34.85	7	20.59	14	35.00	70	30.57	
	Nutritional and endocrine disorder	9	31.03	7	25.00	8	25.00	22	33.33	6	17.65	7	17.50	59	25.76	
**2**	**Non-Communicable diseases**															6.55
	Diseases of the circulatory System	0	0.00	2	7.14	1	3.13	0	0.00	1	2.94	1	2.50	5	2.18	
	Gastrointestinal disorder	0	0.00	1	3.57	1	3.13	1	1.52	2	5.88	0	0.00	5	2.18	
	Mental and nervous system	0	0.00	0	0.00	1	3.13	0	0.00	1	2.94	0	0.00	2	0.87	
	Neoplasm	0	0.00	0	0.00	0	0.00	0	0.00	1	2.94	1	2.50	2	0.87	
	Respiratory disorders	0	0.00	0	0.00	0	0.00	1	1.52	0	0.00	0	0.00	1	0.44	
**3**	**External Causes (Injuries)**															13.10
	External causes of death	2	6.90	4	14.29	3	9.38	3	4.55	9	26.47	9	22.50	30	13.10	
**4**	**Unspecified causes of death**															16.59
	Unspecified	7	24.14	2	7.14	5	15.63	13	19.70	5	14.71	6	15.00	38	16.59	
**5**	**Undetermined Causes of Death**															7.42
	Undetermined	3	10.34	6	21.43	1	3.13	3	4.55	2	5.88	2	5.00	17	7.42	

As it is shown in Table 3, malnutrition, intestinal infectious disease (including diarrhea) and acute lower respiratory infection (including pneumonia) were the leading specific cause of deaths accounting for 33.9%, 13.8% and 9.2% of all deaths, respectively. Considering the year 2013 measles (18.8%) was the second highest specific leading cause of death after malnutrition (21.9%).

**Table 3:** Major specific causes of death by year among children aged 5–14 years, Kersa HDSS, 2008–2013.

Cause_ Specific	Year												Total	
	2008		2009		2010		2011		2012		2013			
	N	%	N	%	N	%	N	%	N	%	N	%	N	%
**Severe malnutrition**	9	47.37	7	35.00	8	30.77	22	44.00	6	22.22	7	21.88	59	33.91
**Intestinal infectious disease including diarrhea**	3	15.79	1	5.00	5	19.23	9	18.00	1	3.70	5	15.63	24	13.79
**Acute lower respiratory infection including pneumonia**	1	5.26	3	15.00	3	11.54	3	6.00	3	11.11	3	9.38	16	9.20
**Measles**	0	0.00	0	0.00	2	7.69	2	4.00	0	0.00	6	18.75	10	5.75
**Meningitis**	0	0.00	0	0.00	0	0.00	5	10.00	3	11.11	0	0.00	8	4.60
**Accidental drowning and submersion**	1	5.26	2	10.00	0	0.00	2	4.00	1	3.70	1	3.13	7	4.02
**Malaria**	3	15.79	1	5.00	1	3.85	1	2.00	0	0.00	0	0.00	6	3.45
**Other transport accidents**	0	0.00	1	5.00	1	3.85	0	0.00	2	7.41	2	6.25	6	3.45
**Assault**	0	0.00	1	5.00	0	0.00	0	0.00	3	11.11	1	3.13	5	2.87
**Congestive heart failure**	0	0.00	2	10.00	0	0.00	0	0.00	1	3.70	1	3.13	4	2.30
**Accidental fall**	0	0.00	0	0.00	0	0.00	0	0.00	0	0.00	3	9.38	3	1.72
**Pedestrian injured in traffic accident**	0	0.00	0	0.00	0	0.00	0	0.00	2	7.41	1	3.13	3	1.72
**Tuberculosis**	1	5.26	1	5.00	0	0.00	1	2.00	0	0.00	0	0.00	3	1.72
**Accident, unspecified**	0	0.00	0	0.00	1	3.85	0	0.00	0	0.00	1	3.13	2	1.15
**Accidental exposure to smoke and fire**	1	5.26	0	0.00	0	0.00	0	0.00	1	3.70	0	0.00	2	1.15
**Acute abdomen**	0	0.00	1	5.00	0	0.00	0	0.00	1	3.70	0	0.00	2	1.15
**Epilepsy**	0	0.00	0	0.00	1	3.85	0	0.00	1	3.70	0	0.00	2	1.15
**Malignant neoplasm of lymphoid and hemopoitic**	0	0.00	0	0.00	0	0.00	0	0.00	1	3.70	1	3.13	2	1.15
**Paralytic ileus and intestinal obstruction**	0	0.00	0	0.00	1	3.85	0	0.00	1	3.70	0	0.00	2	1.15
**Tetanus (excluding tetanus neontoriuum)**	0	0.00	0	0.00	1	3.85	1	2.00	0	0.00	0	0.00	2	1.15
**Accidental poisoning and exposure to venoum**	0	0.00	0	0.00	0	0.00	1	2.00	0	0.00	0	0.00	1	0.57
**Asthma**	0	0.00	0	0.00	0	0.00	1	2.00	0	0.00	0	0.00	1	0.57
**Cerebrovascular diseases**	0	0.00	0	0.00	1	3.85	0	0.00	0	0.00	0	0.00	1	0.57
**Chronic liver disease**	0	0.00	0	0.00	0	0.00	1	2.00	0	0.00	0	0.00	1	0.57
**Contact with unspecified venoum animal or plant**	0	0.00	0	0.00	1	3.85	0	0.00	0	0.00	0	0.00	1	0.57
**Viral hepatitis**	0	0.00	0	0.00	0	0.00	1	2.00	0	0.00	0	0.00	1	0.57
**Total**	19	100.00	20	100.00	26	100.00	50	100.00	27	100.00	32	100.00	174	100.00

Over the entire study period, severe malnutrition, intestinal infectious disease (including diarrhea) and acute lower respiratory infection (including pneumonia) were the three leading causes of death among children aged 5 to 14. Table 4 shows, severe malnutrition accounts for 57 deaths per 100,000 children of age 5 to 14, whereas intestinal infectious disease and acute lower respiratory accounts for 23 and 15 deaths, respectively per 100,000 children of the same age over the study period. Death rate for injuries (including accidents, venom contacts etc.) was 29 per 100,000 children aged 5 to 14 years old.

**Table 4:** Cause Specific Death rates per 100,000 populations for major causes of death by year among children aged 5 to 14, Kersa HDSS, 2008–2013.

Diseases	Year													
	2008		2009		2010		2011		2012		2013		Total	
	N	Rate	N	Rate	N	Rate	N	Rate	N	Rate	N	Rate	N	Rate
**Severe malnutrition**	9	58.556	7	43.085	8	47.148	22	120.153	6	31.687	7	37.926	59	56.57
**Intestinal infectious disease including diarrhea**	3	19.519	1	6.155	5	29.467	9	49.153	1	5.281	5	27.090	24	23.01
**Acute lower respiratory infections including pneumonia**	1	6.506	3	18.465	3	17.680	3	16.384	3	15.844	3	16.254	16	15.34
**Measles**	0	0.000	0	0.000	2	11.787	2	10.923	0	0.000	6	32.508	10	9.59
**Meningitis**	0	0.000	0	0.000	0	0.000	5	27.307	3	15.844	0	0.000	8	7.67
**Accidental drowning and submersion**	1	6.506	2	12.310	0	0.000	2	10.923	1	5.281	1	5.418	7	6.71
**Malaria**	3	19.519	1	6.155	1	5.893	1	5.461	0	0.000	0	0.000	6	5.75
**Other transport accidents**	0	0.000	1	6.155	1	5.893	0	0.000	2	10.562	2	10.836	6	5.75
**Assault**	0	0.000	1	6.155	0	0.000	0	0.000	3	15.844	1	5.418	5	4.79
**Congestive heart failure**	0	0.000	2	12.310	0	0.000	0	0.000	1	5.281	1	5.418	4	3.84
**Accidental fall**	0	0.000	0	0.000	0	0.000	0	0.000	0	0.000	3	16.254	3	2.88
**Pedestrian injured in traffic accident**	0	0.000	0	0.000	0	0.000	0	0.000	2	10.562	1	5.418	3	2.88
**Tuberculosis**	1	6.506	1	6.155	0	0.000	1	5.461	0	0.000	0	0.000	3	2.88
**Accident, unspecified**	0	0.000	0	0.000	1	5.893	0	0.000	0	0.000	1	5.418	2	1.92
**Accidental exposure to smoke and fire**	1	6.506	0	0.000	0	0.000	0	0.000	1	5.281	0	0.000	2	1.92
**Acute abdomen**	0	0.000	1	6.155	0	0.000	0	0.000	1	5.281	0	0.000	2	1.92
**Epilepsy**	0	0.000	0	0.000	1	5.893	0	0.000	1	5.281	0	0.000	2	1.92
**Malignant neoplasm of lymphoid and hemopoitic**	0	0.000	0	0.000	0	0.000	0	0.000	1	5.281	1	5.418	2	1.92
**Paralytic ileus and intestinal obstruction**	0	0.000	0	0.000	1	5.893	0	0.000	1	5.281	0	0.000	2	1.92
**Tetanus (excluding tetanus neontoriuum)**	0	0.000	0	0.000	1	5.893	1	5.461	0	0.000	0	0.000	2	1.92
**Accidental poisoning and exposure to venoum**	0	0.000	0	0.000	0	0.000	1	5.461	0	0.000	0	0.000	1	0.96
**Asthma**	0	0.000	0	0.000	0	0.000	1	5.461	0	0.000	0	0.000	1	0.96
**Cerebrovascular diseases**	0	0.000	0	0.000	1	5.893	0	0.000	0	0.000	0	0.000	1	0.96
**Chronic liver disease**	0	0.000	0	0.000	0	0.000	1	5.461	0	0.000	0	0.000	1	0.96
**Contact with unspecified venoum animal or plant**	0	0.000	0	0.000	1	5.893	0	0.000	0	0.000	0	0.000	1	0.96
**Viral hepatitis**	0	0.000	0	0.000	0	0.000	1	5.461	0	0.000	0	0.000	1	0.96
**UNDT**	3	19.519	6	36.930	1	5.893	3	16.384	2	10.562	2	10.836	17	16.30
**Unspecified causes of death**	7	45.543	2	12.310	5	29.467	13	70.999	5	26.406	6	32.508	38	36.44
**Total**	29		28		32		66		34		40		229	

Table 5 presents the top 10 major cause of death among children aged 5 to 14 during the entire study period. Severe malnutrition, intestinal infectious diseases and acute lower respiratory infections were the top three leading causes of death, respectively. Other important causes of deaths were measles, meningitis and accidental drowning. With very little fluctuation, severe malnutrition remained to be the leading cause of death over the study period.

**Table 5:** Top 10 causes of death among children 5–14 year of age in Kersa HDSS, 2008–2013.

No	Cause_ Specific	Year						Total	
		2008	2009	2010	2011	2012	2013		
								N	%
**1**	Severe malnutrition	9	7	8	22	6	7	59	33.91
**2**	Intestinal infectious disease including diarrhea	3	1	5	9	1	5	24	13.79
**3**	Acute lower respiratory infection including pneumonia	1	3	3	3	3	3	16	9.20
**4**	Measles	0	0	2	2	0	6	10	5.75
**5**	Meningitis	0	0	0	5	3	0	8	4.60
**6**	Accidental drowning and submersion	1	2	0	2	1	1	7	4.02
**7**	Malaria	3	1	1	1	0	0	6	3.45
**8**	Other transport accidents	0	1	1	0	2	2	6	3.45
**9**	Assault	0	1	0	0	3	1	5	2.87
**10**	Congestive heart failure	0	2	0	0	1	1	4	2.30

## Discussions

In this study a total of 229 deaths of children aged 5 to 14 years were reviewed over the entire study period. About six out of seven deaths took place outside health institutions, while less than 15% deaths reported to be at health institutions. The study identified severe malnutrition, intestinal infectious diseases (including diarrhea) and acute lower respiratory infections (including pneumonia) to be the three most leading causes of death in the study population. The finding of this paper showed that majority of deaths occurred among rural residents. During the whole study time, 231 and 120 deaths of children aged 5 to 14 per 100,000 population of the same age group reported for rural and urban, respectively. This result is comparable with the study done elsewhere in Ethiopia which showed that the risk of death for children aged 5 to 14 who lives in rural areas was much higher than those of their urban counterparts[27]. The possible reasons for higher mortality for rural children could be because of disadvantages of rural children in socioeconomic and health care services compared to urban children.

During the study period 219.6 deaths per 100,000 children aged 5 to 14 was reported. Though comparison may be affected a little bit by estimate variation, INDEPTH member HDSS sites like; Matlab (Bangladesh), Bandarban (Bangladesh), Chakaria (Bangladesh), AMK (Bangladesh), Ouagadougou (Burkina Faso), Kilite-Awlaelo (Ethiopia), Dodowa (Ghana), Ballabgarh (India), Kilifi (Kenya), Nairobi (Kenya), Agincourt (South Africa), Africa Centre (South Africa) and FilaBavi (Vietnam) reported less mortality rate of children of the same age group than finding of the current study[5]. However, this finding is comparable with the findings of Fantahun M, and Sacarlal, et al., which showed that this age group had mortality rate ranging from 1 to 4 deaths per 1000 children[27, 28].

From the total deaths in this study about 56.33%, 6.55%, 13.10% were attributed to communicable, non-communicable and injuries, respectively. The remaining were attributed by unspecified and undetermined. The findings of this study are in line with the study done in Mozambique which showed 73.6%, 9.5% and 4% were attributed by communicable, non-communicable and injuries, respectively[18]. This finding is also in line with the study done in India which showed that communicable diseases cause over 196,000 deaths (approximately 60%) in this age group[29]. Injuries have shown increment with little variation as a cause of mortality among children in this age group. Our finding is in line with a study done in Scotland on injuries among this age group which showed that injuries were the leading cause of death during 2000–06[29].

In this study over 75% of the deaths took place outside of the health facility. This percentage is higher than the study carried in Mozambique which was 56%[28]. This shows that even though access to health facilities has been improved over the past couples of years, death at home is still very high which might shed light on the fact that other factors may be more important in defining the pattern of the health seeking behavior in the study area.

Severe malnutrition and intestinal infectious diseases including diarrhea were found to be major causes of death that consistently caused relatively large mortality rates during the study period which shows the fact that communicable diseases continue to be the public health problem in the study population which requires the attention of the policy makers. Deaths due to communicable diseases in this study is about eight fold compared to deaths due to non-communicable diseases. This finding is consistent to the findings of Misganaw et al in which children of age 0 to 14 years were 16 times more likely to die from communicable, prenatal and nutritional conditions compared to other age categories[27]. However, the finding in this study is different from the findings of the study conducted in the United States of America in 2007 in which 87% of deaths among children aged 5–14 year old were due to non-communicable diseases, i.e., unintentional injuries (36%), cancer (15%), birth defects (6%), homicide (6%), and other causes (24%)[30]. This variation could be due to the fact that the two studies took place in different settings which may cause the variation in the socio-economic and health services status of children.

## Conclusions

This study reported a higher overall mortality rate of children aged 5 to 14 for a reason of avoidable causes, treatable infections and preventable accidents. Hence, further research into the factors, prevention and management of infectious diseases including severe malnutrition, intestinal infectious diseases, and acute lower respiratory infections, combined with the new understanding of the burden and distribution of disease among this age children holds the potential to significantly reduce overall mortality. In addition home death is common in the study population which has an implication on the importance of surveillance data for health sector planning as it provides information on causes of death for those who die at home. Hence, strengthening the activities of the surveillance system is required to get reliable data for the health planning of the study community. And finally, in this study, injuries were reported to be among the top three leading broad causes of death. Thus further study is warranted to identify the associated factors which may lead for targeting preventive efforts.
